# Advanced practice radiation therapist led vaginal vault brachytherapy: An evaluation of efficiency and effectiveness of service delivery

**DOI:** 10.1016/j.tipsro.2024.100289

**Published:** 2024-11-27

**Authors:** Rhona Goodwin

**Affiliations:** University Hospital Galway, Radiation Oncology Department, Newcastle Rd., Galway H91YR71, Ireland

**Keywords:** Advanced practice, Radiation therapist, Stage 1 endometrial carcinoma, Vaginal vault brachytherapy, Efficiency, Recurrence

## Abstract

**Aims:**

An advanced practice radiation therapist (APRT) role in vaginal vault brachytherapy (VBT) was first introduced in 2015. The aim of this manuscript was to clarify how the introduction of an APRT can improve efficiencies within service delivery while maintaining non inferior clinical outcomes of recurrence.

**Materials and methods:**

This was a single-centred retrospective comparative service evaluation of stage 1 endometrial cancer patients treated with postoperative high dose rate VBT alone. Sixty patients in total were included in the evaluation. They were divided into two equal groups of ‘before’ and ‘after’ based on the introduction of the APRT-led role and changes made to treatment processes. Quantitative analysis of waiting times from surgery to adjuvant VBT (weeks), duration of day one treatment procedure (minutes), rate of isolated vaginal recurrence and loco regional recurrence (vaginal and pelvic) were compared between the groups.

**Results:**

The mean time from surgery to treatment between the ‘before’ and ‘after’ groups was 13.1 and 9.1 weeks respectively. The mean time for day one treatment procedure was 91 min in the ‘before’ group and 59 min in the ‘after’ group. Vaginal free recurrence (VFR) was 100 % for both groups..

**Conclusion:**

An APRT led vaginal vault service, with learnt role extension, allows for a more efficient and effective service delivery.

## Highlights

• Upskilling of RTs to APRT roles has an impact on efficiency of service delivery.
• The implementation of APRT roles facilitates change to current processes.
• APRT roles improve quality of patient care.

## Introduction

Endometrial cancer is the most common gynaecological cancer, with 80 % diagnosed as stage 1, in developed countries [1]. The recent average annual incidence of uterine cancers diagnosed in Ireland is 557, making it the most common gynaecological cancer in women in Ireland with a 5-year survival rate of 78 % when diagnosed early [2].

A total abdominal hysterectomy is the primary treatment [1]. Vaginal brachytherapy (VBT) as an adjuvant treatment modality is one option recommended by the 2009 International Federation of Gynaecology and Obstetrics (FIGO) staging system [3]. High risk features for recurrence [4] such as age, grade, depth of myometrial invasion (MMI), and lymph vascular invasion (LVSI) inform risk stratification (Appendix A1, A2, A3). The effectiveness of VBT as a treatment modality in managing vaginal free recurrence (VFR) has been demonstrated in pivotal trials over 20 years [5], [6] and validated further in recent studies [7], [8]. As a result of its efficacy and reduced potency specific to treatment side effects [5] an increased number of patients are being managed with VBT alone as the treatment modality of choice for early stage endometrial carcinoma.

The Radiation Oncology centre this study was based on observed significant delays on the days that a vaginal brachytherapy procedure was booked. Delays were multifactorial but fundamentally due to the lack of dedicated staff to brachytherapy on a day when a case was scheduled. Staff comprised of a multidisciplinary team including the radiation oncologist, radiation therapists, physicists and nurses. Recognising that the VBT treatment process is complex and time consuming due to the co-ordination of independent tasks by each profession involved, a review of our current workflow was required.

In Canada, a pilot study [9] demonstrated that implementation of advanced practice radiation therapist (APRT) roles led to improved service delivery by reducing wait times and increasing throughput of patients. The evidence base within the Irish landscape on advanced practice in vaginal vault brachytherapy was non-existent. A radiation therapist commenced an advanced practice MSc alongside in-house training led by the Radiation Oncologist (RO) and began reviewing our current practices more closely.

The objective of this study was to measure and explore the value of the changes implemented and to communicate the findings at a local level with departmental presentations, at an educational level with colleges and with the wider radiotherapy community at national and international conferences. The work aims to generate evidence to justify advanced practice roles and their impact on efficiency and effectiveness of service delivery within the Irish context.

### Role of the APRT and changes to workflow

**Fig. 1:**
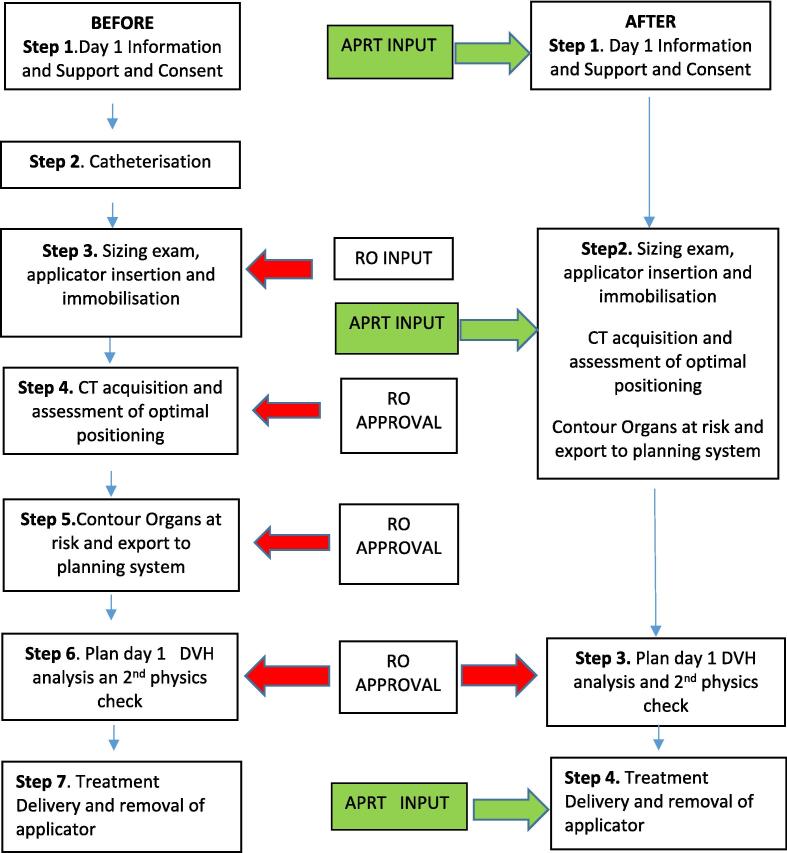
Before’ and ‘after’ patient workflow, demonstrating new streamlined process with reduced need for Radiation Oncologist (RO) approval.

A clinical portfolio alongside academic modules on the theory of brachytherapy, vaginal vault brachytherapy, leadership, change management and research methodology was academically accredited by Queen Margaret University in Edinburgh and encompassed the four pillars of advanced practice. (Appendix B) Upon completion of the vaginal vault clinical practice module, the RT was upskilled on interpreting histopathology’s, understanding appropriate treatment management decisions, vaginal speculum examinations including sizing examinations for appropriate applicator selection, applicator positioning and contouring the organs at risk. A second radiation therapist (RT) began the same academic and clinical practice MSc to facilitate growth of the radiation therapist brachytherapy team. The aim of upskilling the radiation therapist was to implement an advanced practice role dedicated to brachytherapy, expanding the role and responsibilities in order to reduce RO input, including task approval, to facilitate a more efficient workflow. (Fig. 1) The RT was introduced to outpatient clinics and shadowed new and follow up consultations throughout the MSc and in now actively participating in clinic on a weekly basis to consolidate the theoretical knowledge and build a rapport with patients before attending for treatment and in the follow up setting.

Fig. 1 demonstrates that catheterisation has been omitted in the ‘after’ workflow. From attending brachytherapy workshops and conferences it was evident that the need for catheterisation had become outdated. Historically, the role of the balloon pulled back to the base of the bladder upon catheterisation facilitated the creation of the International Commission on Radiation Units and measurement, 1985 (ICRU) point for estimating bladder dose. With 3D planning to assess bladder dose omitting catheterisation was the first change in the patient workflow instigated by the APRT.

## Materials and methods

This evaluation set out to establish its effectiveness by identifying key questions. All the answers were objective and available through our patient electronic database, MOSAIQ**®**. Evaluation objectives were defined using SMART. Table 1 [10].

**Table 1:** Evaluation Objectives defined by SMART.

**Objective 1** − Efficiency
**S**pecific − Efficiency of referral process **M**easurable – Time from surgery to treatment **A**ttainable – Existing data − using patient oncology treatment database **R**elevant – Best practice guidelines 12 weeks **T**ime – Before and after APRT role introduction to the VBT service
**Objective 2**- Efficiency
**S**pecific − Efficiency day 1 treatment process **M**easurable – Time from applicator insertion (CT scanning) to removal (after treatment) **A**ttainable – Existing data − using patient oncology treatment database **R**elevant – Efficient use of CT, efficient use of staff and patient comfort **T**ime – Before and after APRT role introduction to the VBT service
**Objective 3** − Effectiveness
**S**pecific – Effectiveness of clinical outcome **M**easurable – % Recurrence rates **A**ttainable – Existing data − using patient oncology treatment database **R**elevant – Change needs to ensure no negative impact on patient outcomes **T**ime – Before and after APRT role introduction to the VBT service

### Study population

**Fig 2:**
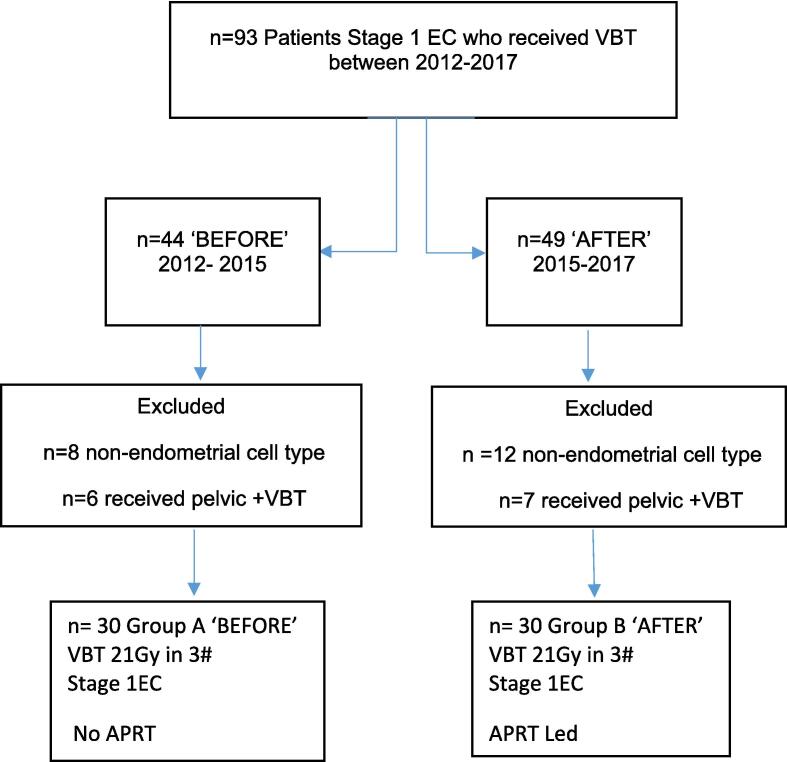
Study Flow chart. Vaginal brachytherapy (VBT), Endometrial carcinoma (EC), Advanced practice radiation therapist (APRT).

All patients included in the evaluation were surgically stage 1 as per FIGO classification (Appendix A1). All patients received VBT alone between January 2012 and October 2017. Patients who received chemotherapy, pelvic radiotherapy or nonstandard VBT were excluded. Patients with non-endometroid cancers (serous, clear cell, papillary or mixed) were also excluded. Patients were allocated into ‘before’ and ‘after’ groups, based on the implementation of the APRT, as shown in the study flowchart below (Fig. 2).

### Brachytherapy

VBT was delivered using a Varian single channel cylinder, ranging in size from 2.0 cm to 3.5 cm dependent on patient anatomy. The selection of the cylinder size was based on a clinical examination of the vaginal vault. The applicator was immobilised with the Varian^R^ universal clamping device. A CT scan to aid planning was acquired using a Toshiba AQUILION 16 scanner. The treatment planning system, Brachyvision^TM^, assessed target coverage and dose to organs at risk. The standard dose delivered was 21 Gy in three fractions to the proximal 4 cm of the vaginal vault prescribed to 5 mm depth from the surface of the cylinder.

### Follow up

Follow up consisted of four weeks after completing treatment, then every three months for the first year with care alternating between the gynaecology team and the radiation therapy team. After year one, appointments were every six months until year three then yearly to year five. Patients were discharged at 60 months where there was no evidence of symptom toxicity or recurrence.

### Data Collection and statistical analysis

Eligible patients were selected from the patient electronic radiotherapy treatment database, MOSAIQ**®**. Each individual patient’s electronic chart was reviewed for measureable specific data as defined in Table 1. All patient files were analysed and assessed for diagnosis of recurrence up to a maximum of 60 months from the date of their surgery. All data collected was meticulously entered into a statistical package for extrapolation of results.

Data was managed using an Excel database and statistically analysed using IBM SPSS Statistics for windows, version 29.0 [11]. A P-value of < 0.05 was considered statistically significant. Descriptive statistics allowed statistical significances to be visualised. Independent t-tests were performed to compare means between the groups for continuous variables of waiting time to adjuvant VBT and time of day one treatment procedure. Locoregional recurrence free survival was determined using the Kaplan- Meier method. Patients were censored at the time of first recurrence, 60 month follow up, or the last recorded follow up prior to 60 months.

### Ethical approval

An application for ethics approval was submitted to the research ethics committee at University Hospital Galway. Formal ethics approval was not required due to the methodology of the study defining it as a service evaluation. All patient data was anonymised.

## Results

Sixty patients in total were included in the analysis. Thirty received adjuvant VBT alone before the introduction of an APRT to the VBT service and 30 received treatment after. The characteristics of the whole study population are reported in Table 2. The median age of the women was 64 and 65.7 respectively between the groups. On comparison of the ‘before’ and ‘after’ groups, 23.3 % and 20 % had FIGO 1a disease respectively with 76.7 % and 80 % diagnosed as FIGO 1b. The majority of patients had G1-G2 disease in the ‘after’ group (96.7 %) with only 3.3 % having G3. Forty per cent of patients in the ‘before’ group had G3 disease. Patients were categorised into risk group, as outlined in Appendix A3, according to the European Society for Medical Oncology- European society for gynaecological oncology- European society for radiotherapy and oncology (ESMO-ESGO-ESTRO) consensus guidelines [1].

**Table 2:** Patient characteristics of whole study population.

Characteristics		Before n (%)	After n (%)
Patients		30	30
Median age (range)		64 (47–89)	65.7 (55–80)
FIGO Stage	1a	7 (23.3 %)	6 (20 %)
	1b	23 (76.7 %)	24 (80 %)
Histology	Endometrioid	30 (100 %)	30 100 %)
Grade	1 2 3	7 (23.3 %) 11 (36.7 %) 12 (40 %)	11 (36.7 %) 18 (60 %) 1 (3.3 %)
LVSI	Absent Present Unspecified	26 4 −	18 12 −
Risk classification *	Intermediate High-Intermediate High	16 (53.4 %) 2 (6.6 %) 12 (40 %)	18 (60 %) 11 (36.7 %) 1 (3.3 %)

FIGO 2009:International Federation of Obstetrics Gynaecology; LVSI: lymphovascular space invasion *Risk classification as per European Society for Medical Oncology- European society for gynaecological oncology- European society for radiotherapy and oncology (ESMO-ESGO-ESTRO).

### Waiting times from surgery to VBT

**Fig. 3:**
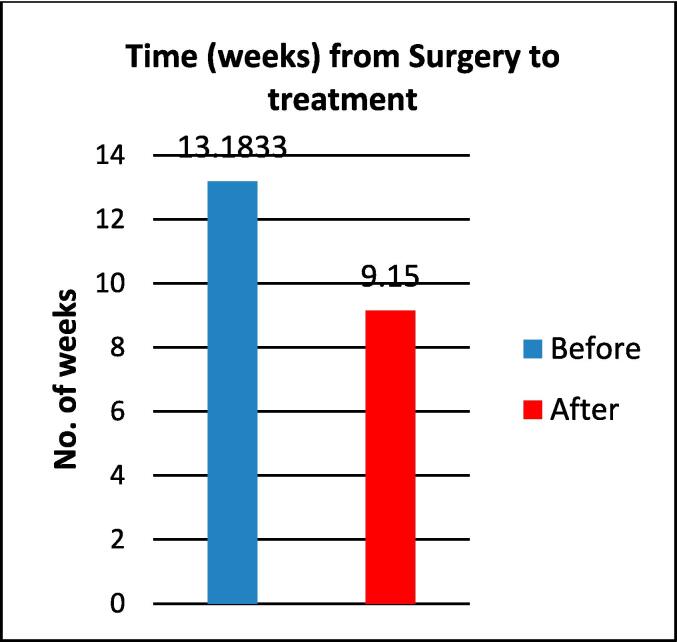
Time (weeks) from surgery to VBT alone for ‘Before’ and ‘After’ groups.

**Fig. 4:**
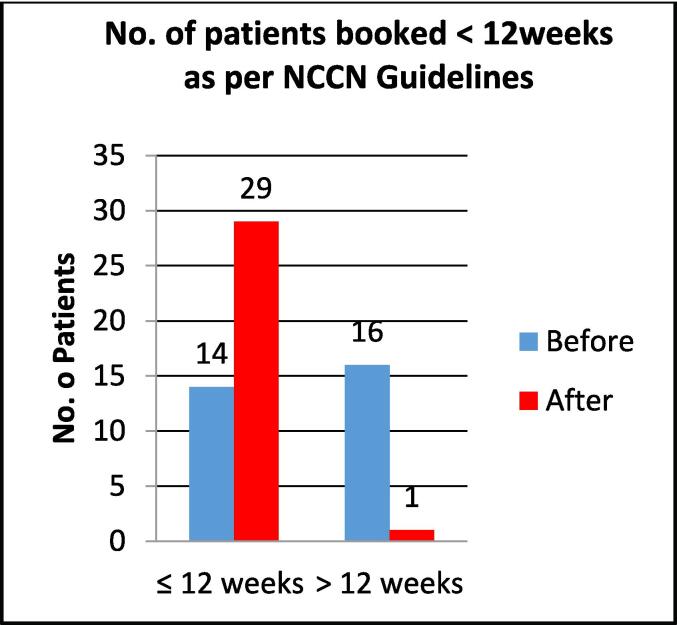
Number of patients booked within best practice guidelines as per NCCN guidelines.

The average waiting time for the ‘before’ and ‘after’ study population from surgery to treatment was 13.18 weeks and 9.15 weeks respectively (Fig. 3). An independent *t*-test demonstrates the statistical significance of this change with a P-value of < 0.001. Fifty three per cent (n = 16) of the ‘before’ group were treated outside of best practice guidelines which is defined as 12 weeks post-surgery once the vaginal cuff has healed [12]. In the ‘after’ group 97 % (n = 29) of patients fell within the best practice guidelines with one case 3 % (n = 1), being delayed due to misplaced referral (Fig. 4).

### Duration of day 1 workflow

The mean time the applicator was positioned in the vaginal vault on day one was 91 min in the ‘before’ group and 59 min in the ‘after’ group. An independent *t*-test demonstrates the statistical significance of this change with a P-value of < 0.001. The minimum, maximum and mean times are presented in Table 3.

**Table 3:** Time of day 1 procedure for ‘Before’ and ‘After’ groups in minutes.

Time	Before APRT (min)	After APRT(min)
Minimum	75	45
Maximum	150	90
Mean	91	59

**Fig. 5:**
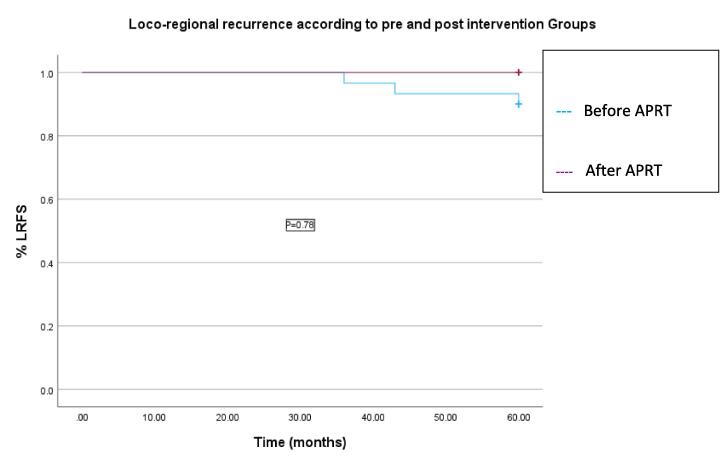
Kaplan Meier chart demonstrating overall locoregional recurrence free survival (LRFS) between the ‘before’ and ‘after’ groups.

All patients were followed up for 60 months, with this taken as the cut off point for analysis of recurrence. Locoregional recurrence (vaginal or pelvic) was seen in 2/30 (6.6 %) with synchronous pulmonary recurrence and an additional 1/30 (3.3 %) had distant recurrence alone (pulmonary) in the ‘before’ group. There was 100 % VFR in both groups and no locoregional recurrence in the ‘after’ group. One patient died 2 years after treatment. She was 82 years old and cause of death was unrelated to diagnosis. Patient characteristics including site and timing of recurrence are reported in Table 4 with time to event of recurrence demonstrated in Fig. 5.

**Table 4:** Recurrent population characteristics.

Group	Before	Before	Before
FIGO Stage	1b	1b	1b
Risk category	High	Intermediate	Intermediate
Grade	G3	G2	G2
LVSI	No	No	No
Time to treatment (Weeks)	12	10.5	19
Loco Regional recurrence (Site)	Pelvis, Lung	Pelvis, Lung	Lung
Time to recurrence (Months)	43	60	36

All statistical analysis can be found in Appendix C.

## Discussion

Treating patients within best practice guidelines is fundamental to high quality patient care. Guidelines recommend treatment when the vaginal cuff has healed and within 12 weeks of Surgery [12]. When we look at our data in the ‘before’ group we can see significant delays from surgery to treatment (Fig. 3). Although the reasons behind delays were not explored in this evaluation, it is evident from practice that awaiting radiological investigations, MDM discussions and private referrals can be contributing factors. Interestingly, Neron et al. [13]. investigated the cause of delay and found that both the surgical route and delays awaiting MDM were statistically significant (P = 0.009). With the dedicated brachytherapy APRT central to the patient management from MDM discussion a significant statistical difference (P < 0.001) of waiting times between the ‘before ‘and ‘after’ groups has been achieved (Fig. 3). Empowered with education and key information, including surgical dates, forward planning and coordination of the brachytherapy timetable was possible and facilitated a positive change.

While research has attempted to address the association between delays and recurrence, many of the studies have methodological flaws that limit their validity [14], [15], [16]. The most recent study to show a statistical significance with delays to treatment and recurrence was Zhu et al. [14]. Patients who received adjuvant radiation therapy within 8 weeks experienced significantly less disease recurrence (9 % vs 18 %; p = 0.01) and less isolated vaginal recurrence (0 % vs 6 %, p = 0.04). With 83 % of Zhu’s study population diagnosed as FIGO stage 1 and 58 % of these receiving VBT alone, this study was the closest for purposes of comparison and validation with our results. However, there is no standardisation between treatment techniques, doses and fractionation regimes over the 30 year timeframe of the study, which is in total contrast to our evaluation with all patients treated with the same technique and dose over a relatively short timeframe. Cattaneo et al. [15] demonstrated a 5-year recurrence free survival of 90 % in those treated < 9 weeks and 39 % > 9 weeks (P = 0.001). Ambiguity exists for comparison with this evaluation, not only by inclusion of patients with non-endometrial cell type and advanced disease but with only 29 % of patients receiving VBT. Consistent with the cut-off point of 9 weeks implemented by Cattaneo et al. [15]. Fabrini et al. [16] also demonstrated a statistical significance in association of delays to treatment > 9 weeks (P = 0.046). What stands out as significant on comparison with our results, is that all recurrence presented in patients treated > 9 weeks from surgery (Table 4). Pivotal randomised trials [5], [6] in the validation of VBT as an appropriate treatment modality implemented 8 weeks as the cut-off point when assessing recurrence. Although very small numbers were compared in this evaluation, the value of reducing waiting times from our results demonstrates a positive impact on clinical outcome of recurrence with 100 % LRFS versus 93.4 % when using 8 weeks as the cut-off point as per pivotal trial protocol [5], [6] or 9 weeks as implemented in the two studies discussed above [15], [16].

High quality patient care expands beyond treating within best practice timelines. From our clinical experience we know that each patient presents with different needs. From a theoretical standpoint we can ensure a high quality treatment plan and deliver it safely. However, patient centred care encompasses the entire patient’s physiological wellbeing. The impact of waiting times on the patient’s psychological wellbeing is widely recognised in literature [17], [18]. One study by Park et al. [19] examined anxiety in patients specific to VBT treatment. He concluded that up to 70 % of women undergoing VBT treatment suffer with anxiety related to fear and pain of the treatment. Prioritising patient comfort meant analysing our workflow to reduce the length of time the vaginal applicator remained in the vaginal vault.

Although limited, there was evidence by both Paterson et al. [20] and Nahab et al. [21] to support an efficient RT led VBT service. Despite Paterson et al. [20] lacking statistical quantifiable data, as its discussion focused on implementation of the role, it still provides evidence of RT efficiency in the implementation process. Nahab et al. [21] on the other hand provided quantitative results of minimum, maximum and average times before and after streamlining their process, consistent with this evaluation. Delegation of tasks traditionally completed by one professional group to another is one approach to improve continuity of service delivery [22]. This was the approach evident from both manuscripts and essentially what has been untaken in our department. The removal of waiting for RO approval at a number of steps in the process (Fig. 1) streamlines the patient pathway reducing the length of time the applicator is in the vaginal vault (Table 3) and ultimately how long the patient is in the department. This has a direct impact on patient centred care especially specific to pain management as well as flexibility of service delivery. Reducing the need for the RO also increases departmental efficiency as the consultant is not required to leave clinic at various points in the process, facilitating punctual clinics, punctual CT scanning schedule and punctual throughput of vaginal brachytherapy treatment procedures.

None of the data presented on improvements in efficiency are significant unless they are backed up with successful outcomes of recurrence. One of the pivotal trials validating the role of VBT was the PORTEC 2 trial [5]. The study population included high intermediate risk (HIR) patients as classified by the PORTEC-1 trial (See Appendix A2) [6]. The results of recurrence from our evaluation were comparable with PORTEC 2 [5], with 0 % and 1.8 % isolated vaginal recurrence respectively and identical locoregional recurrence of 5 % (3/60/). However, despite the similarity, it is important to note the significance of changes in risk stratification over time when comparing results. We found that 61.1 % of the patients in our evaluation fell into the HIR as per PORTEC risk classification. However, when re stratified as per most recent ESMO-ESGO-ESTRO guidelines [1] only 21.6 % (13/60) were categorised as HIR meaning that a significant proportion, 56.6 % (34/60) of the study population now fell into a lower risk category of recurrence. (Appendix A3) Buckley et al. [23] utilising the ESMO-ESGO-ESTRO guidelines for risk stratification, reported 2.2 % isolated vaginal recurrence post VBT alone and 3.6 % locoregional recurrence. Thirty seven percent of the patients were HIR and 100 % of the study population were treated with VBT. Our results of 0 % vaginal recurrence and 5 % locoregional recurrence are in line with both pivotal trials and more recent data. This study had further relevance to ours as it is the only published study on clinical outcomes of recurrence with an RT led VBT service [23].

## Conclusion

The fundamental aim for all cancer services is to provide evidence-based care that is effective, safe, of high quality and patient-centred [24]. This evaluation, through improved efficiencies encompassing best practice guidelines and patient comfort, demonstrates high quality patient centred care. The effectiveness of the treatment in achieving local recurrence rates in line with international data validates the APRT role. The robustness of this evaluation is evident as it demonstrates why and how the role was implemented as well as showing its benefits. The success is evident as it is embedded into normal practice within our department since 2015 and is continually evolving. Further changes to processes, to alleviate fear and anxiety for the patient associated with brachytherapy at our institution are underway, ensuring continuity of care, dedicated education and support sessions on brachytherapy and telephone consultations in the aftercare setting. Evaluation of these change processes will form the foundation of further research.

## Funding

This research did not receive any specific grant from funding agencies in the public, commercial, or not-for-profit sectors.

## Declaration of competing interest

The authors declare that they have no known competing financial interests or personal relationships that could have appeared to influence the work reported in this paper.

## Figures and Tables

**Table:** 

FIGO Stage	
Ia	Tumor confined to the corpus uteri
IAa	No or less than half myometrial invasion
IBa	Invasion equal to or more than half of the myometrium
IIa	Tumor invades cervical stroma, but does not extend beyond the uterus
IIIa	Local and/or regional spread of the tumor
IIIAa	Tumor invades the serosa of the corpus uteri and/or adnexae
IIIBa	Vaginal involvement and/ or parametrial involvement
IIICa	Metastases to pelvic and/or *para*-aortic lymph node
IIIC1a	Positive pelvic nodes
IIIC2a	Positive *para*-aortic nodes with or without positive pelvic lymph nodes
IVa	Tumor invades bladder and/or bowel mucosa, and/or distant metastases
IVAa	Tumor invasion of bladder and/or bowel mucosa
IVBa	Distant metastasis, including intra-abdominal metastases and/or inguinal nodes)

**Table:** 

	PORTEC-1	GOG 99
Age	60	See below
Grade	G 3	G 2–3
Myometrial invasion	50 %	66.6 % (outer 1/3)
Lymphovascularspace invasion	N/A	Present
High-intermediaterisk group	At least 2/3 above	Any age, all 3 of aboverisk factors; ageO50,2 of above risk factors;Age > 70, 1 of aboverisk factors

**Table:** 

Risk group	Description
Low	Stage I endometrioid, grade 1–2, <50 % myometrial invasion, LVSI negative
Intermediate	Stage I endometrioid, grade 1–2, ≥50 % myometrial invasion, LVSI negative
High-intermediate	Stage I endometrioid, grade 3, <50 % myometrial invasion, regardless of LVSI status
	Stage I Endometrioid, grade 1–2, LVSI unequivocally positive, regardless of depth of invasion
High	Stage I endometrioid, grade 3, ≥50 % myometrial invasion, regardless of LVSI statusStage IIStage III endometrioid, no residual diseaseNon endometrioid (serous or clear cell or undifferentiated carcinoma, or carcinosarcoma)
Advanced	Stage III residual disease and stage IVA
Metastatic	Stage IVB

**Table:** 

1	Understand current clinical practice and management of patients with endometrial cancer locally and internationally and how these align	Contains a section outlining how the Institution's clinical protocol aligns with the literature.Compulsory readings *See below
2	Understand current expert body recommendations	Contains a summary of the American Brachytherapy Society (ABS) and Groupe Européen de Curiethérapie and the European Society for Radiotherapy & Oncology (GEC-ESTRO) recommendations on VBT.Compulsory readings: **See below
3	Understand female pelvic anatomy before and after surgery	Describes surgical pathway based on disease stage and grade at our Institution.Contains anatomical diagrams and CT images of female pelvic anatomy pre and post-surgery.
4	Understand possible side effects and ways in which these can be managed Demonstrate understanding of when to refer to ROb. Side effects related to brachytherapy and surgery	Covers reported rates of treatment related side-effects.Describes the symptoms, causes, assessment, management and referral criteria for various side-effects: • Bladder • Bowel (diarrhoea and constipation) • Vaginal • Infection • Pain • Prolapse • Menopause
5	Understand the importance of correct size and placement of applicator	Covers information on the range of applicators available (locally and internationally), effect of air gaps, reports on incorrect treatment delivery in cylinder based VBT and describes the insertion process. Beneficial Reading, not compulsory *** **See below**
6	Successful completion of 20 vaginal cylinder insertions under direct RO guidance and supervision	The RO will perform the first insertion and describe their technique to the RT. The RT will then perform the subsequent insertions for that patient. It is therefore anticipated that the RT will have experience performing cylinder insertions on a minimum of ten patients prior to sign-off.
8	Complete a reflective case study for each training insertion	Reflective case study template created based on Gibbs’ Reflective Cycle
9.	On completion of MSc with advanced knowledge and 2 years experience supported by RO	The APRT will perform first insertion and sizing examinations as signed off by the RO

**Table:** 

**Name**	**Type**	**Label**	**Measure**	**Role**
Group	Numeric	Group A(pre) or Group B (post)	Nominal	input
Time To Treatment	Numeric	Weeks Surgery to adjuvant VBT	Scale	input
Time applicator insitu	Numeric	Minutes applicator in vaginal vault	Scale	input
Age	Numeric	Years	Scale	input
Stage	Numeric	Figo 2009	Nominal	input
Grade	Numeric	Histopathological Grade	Nominal	input
Risk	Numeric	ESMO-ESGO risk stratification	Nominal	input
Recurrence	Numeric	Locoregional recurrence	Nominal	input
Time to Recurrence	Numeric	Months	Scale	input

**Table:** 

Group A (before)	N	Valid	30
		Missing	0
	Mean		13.1833
	Median		12.5000
	Std. Deviation		3.95771
	Range		18.00
	Percentiles	25	10.8750
		50	12.5000
		75	16.0000
Group B (after)	N	Valid	30
		Missing	0
	Mean		9.1500
	Median		9.0000
	Std. Deviation		1.77701
	Range		9.00
	Percentiles	25	8.0000
		50	9.0000
		75	10.0000

**Table:** 

Group A (before) or Group B (after)			Frequency	Percent	Valid Percent	Cumulative Percent
Group A (before)	Valid	6.00	2	6.7	6.7	6.7
		8.50	1	3.3	3.3	10.0
		10.00	3	10.0	10.0	20.0
		10.50	1	3.3	3.3	23.3
		11.00	3	10.0	10.0	33.3
		12.00	4	13.3	13.3	46.7
		12.50	3	10.0	10.0	56.7
		13.00	3	10.0	10.0	66.7
		14.00	1	3.3	3.3	70.0
		16.00	3	10.0	10.0	80.0
		17.00	1	3.3	3.3	83.3
		18.00	2	6.7	6.7	90.0
		19.00	2	6.7	6.7	96.7
		24.00	1	3.3	3.3	100.0
		Total	30	100.0	100.0	
Group B (after)	Valid	7.00	2	6.7	6.7	6.7
		7.50	2	6.7	6.7	13.3
		8.00	9	30.0	30.0	43.3
		9.00	5	16.7	16.7	60.0
		9.50	2	6.7	6.7	66.7
		10.00	6	20.0	20.0	86.7
		10.50	1	3.3	3.3	90.0
		11.00	1	3.3	3.3	93.3
		12.00	1	3.3	3.3	96.7
		16.00	1	3.3	3.3	100.0
		Total	30	100.0	100.0	

**Table:** 

Group A (Before)	N	Valid	30
		Missing	0
	Mean		91.5000
	Median		90.0000
	Std. Deviation		19.61483
	Range		90.00
	Percentiles	25	80.0000
		50	90.0000
		75	90.0000
Group B (after)	N	Valid	30
		Missing	0
	Mean		58.8333
	Median		60.0000
	Std. Deviation		11.11719
	Range		50.00
	Percentiles	25	50.0000
		50	60.0000
		75	60.0000

**Table:** 

Group A (before) or Group B (after)		N	%
Group A (before)	60.00	2	6.7 %
	70.00	1	3.3 %
	75.00	1	3.3 %
	80.00	7	23.3 %
	90.00	13	43.3 %
	120.00	5	16.7 %
	150.00	1	3.3 %
Group B (after)	40.00	1	3.3 %
	45.00	4	13.3 %
	50.00	3	10.0 %
	55.00	1	3.3 %
	60.00	17	56.7 %
	70.00	2	6.7 %
	90.00	2	6.7 %

**Table:** 

**Group Statistics**					
	Subject	N	Mean	Std. Deviation	Std. Error Mean
Weeks from Surgery to adjuvant VBT	Group A (before)	30	13.1833	3.95771	0.72258
	Group B (after)	30	9.1500	1.77701	0.32444

**Table:** 

		Levene's Test for Equality of Variances		*t*-test for Equality of Means
		F	Sig.	t
Weeks from Surgery to adjuvant VBT	Equal variances assumed	11.922	0.001	5.092
	Equal variances not assumed			5.092
**Independent Samples Test**				
			*t*-test for Equality of Means	
			Significance	
		df	One-Sided p	Two-Sided p
Weeks from Surgery to adjuvant VVBT	Equal variances assumed	58	0<.001	0<.001
	Equal variances not assumed	40.236	0<.001	0<.001

**Table:** 

**Independent Samples Test**			
		*t*-test for Equality of Means	
		Mean Difference	Std. Error Difference

Weeks from Surgery to adjuvant VBT	Equal variances assumed	4.03333	0.79207
	Equal variances not assumed	4.03333	0.79207

Independent Samples Test			
		*t*-test for Equality of Means	
		95 % Confidence Interval of the Difference	
		Lower	Upper

Weeks from Surgery to adjuvant VBT	Equal variances assumed	2.44783	5.61883
	Equal variances not assumed	2.43279	5.63387

**Table:** 

Group Statistics					
	Group A or Group B	N	Mean	Std. Deviation	Std. Error Mean
Minutes applicator in vaginal vault	Group A (before)	30	91.5000	19.61483	3.58116
	Group B (after)	30	58.8333	11.11719	2.02971

**Table:** 

**Independent Samples Test**				
		Levene's Test for Equality of Variances		*t*-test for Equality of Means
		F	Sig.	t
Minutes applicator in vaginal vault	Equal variances assumed	4.559	0.037	7.936
	Equal variances not assumed			7.936

**Table:** 

**Independent Samples Test**				
		*t*-test for Equality of Means		
			Significance	
		Df	One-Sided p	Two-Sided p
Minutes applicator in vaginal vault	Equal variances assumed	58	0<.001	0<.001
	Equal variances not assumed	45.889	0<.001	0<.001

**Table:** 

**Independent Samples Test**			
		*t*-test for Equality of Means	
		Mean Difference	Std. Error Difference
Minutes applicator in vaginal vault	Equal variances assumed	32.66667	4.11636
	Equal variances not assumed	32.66667	4.11636

**Table:** 

**Independent Samples Test**			
		*t*-test for Equality of Means	
		95 % Confidence Interval of the Difference	
		Lower	Upper
Minutes applicator in vaginal vault	Equal variances assumed	24.42687	40.90646
	Equal variances not assumed	24.38032	40.95302

**Table:** 

Group A (before) or Group B (after)	Total N	N of Events	Censored	
			N	Percent
Group A (before)	30	3	27	90.0 %
Group B (after)	30	0	30	100.0 %
Overall	60	3	57	95.0 %

**Table:** 

Group A (Pre)	Time	Status	Estimate	Std. Error	N. of Cumulative Events	N. of Remaining cases
1	36.00	Recurrence	.967	.033	1	59
2	43.00	Recurrence	.933	.046	2	28
3	60.00	Recurrence	.900	.055	3	27
4	60.00	No recurrence	-	-	3	26
5	60.00	No recurrence	-	-	3	25
6	60.00	No recurrence	-	-	3	24
7	60.00	No recurrence	-	-	3	23
8	60.00	No recurrence	-	-	3	22
9	60.00	No recurrence	-	-	3	21
10	60.00	No recurrence	-	-	3	20
11	60.00	No recurrence	-	-	3	19
12	60.00	No recurrence	-	-	3	18
13	60.00	No recurrence	-	-	3	17
14	60.00	No recurrence	-	-	3	16
15	60.00	No recurrence	-	-	3	15
16	60.00	No recurrence	-	-	3	14
17	60.00	No recurrence	-	-	3	13
18	60.00	No recurrence	-	-	3	12
19	60.00	No recurrence	-	-	3	11
20	60.00	No recurrence	-	-	3	10
21	60.00	No recurrence	-	-	3	9
22	60.00	No recurrence	-	-	3	8
23	60.00	No recurrence	-	-	3	7
24	60.00	No recurrence	-	-	3	6
25	60.00	No recurrence	-	-	3	5
26	60.00	No recurrence	-	-	3	4
27	60.00	No recurrence	-	-	3	3
28	60.00	No recurrence	-	-	3	2
29	60.00	No recurrence	-	-	3	1
30	60.00	No recurrence	-	-	3	0
Group B (Post)	Time	Status	Estimate	Std. Error	N. of Cumulative Events	N. of Remaining cases
1	60.00	No recurrence	-	-	0	29
2	60.00	No recurrence	-	-	0	28
3	60.00	No recurrence	-	-	0	27
4	60.00	No recurrence	-	-	0	26
5	60.00	No recurrence	-	-	0	25
6	60.00	No recurrence	-	-	0	24
7	60.00	No recurrence	-	-	0	23
8	60.00	No recurrence	-	-	0	22
9	60.00	No recurrence	-	-	0	21
10	60.00	No recurrence	-	-	0	20
11	60.00	No recurrence	-	-	0	19
12	60.00	Death	-	-	0	18
13	60.00	No recurrence	-	-	0	17
14	60.00	No recurrence	-	-	0	16
15	60.00	No recurrence	-	-	0	15
16	60.00	No recurrence	-	-	0	14
17	60.00	No recurrence	-	-	0	13
18	60.00	No recurrence	-	-	0	12
19	60.00	No recurrence	-	-	0	11
20	60.00	No recurrence	-	-	0	10
21	60.00	No recurrence	-	-	0	9
22	60.00	No recurrence	-	-	0	8
23	60.00	No recurrence	-	-	0	7
24	60.00	No recurrence	-	-	0	6
25	60.00	No recurrence	-	-	0	5
26	60.00	No recurrence	-	-	0	4
27	60.00	No recurrence	-	-	0	3
28	60.00	No recurrence	-	-	0	2
29	60.00	No recurrence	-	-	0	1

**Table:** 

**Overall Comparisons**			
	Chi-Square	df	Sig.
Log Rank (Mantel-Cox)	3.105	1	0.078
Test of equality of survival distributions for the different levels of Group A (pre) or Group B (post).			

## Appendix A **−** staging and risk stratification tables

### A1. Cancer of the corpus uteri FIGO staging.

FIGO International Federation of Gynecology and Obstetrics;

a Either G1, G2, or G3.b Endocervical glandular involvement only should be considered as Stage I and no longer as Stage II.c Positive cytology has to be reported separately without changing the stage.

A2. High-intermediate risk group as defined by PORTEC-1 and GOG 99.FIGO International Federation of Gynecology and Obstetrics;

PORTEC 5 Post-Operative Radiation Therapy in Endometrial Cancer;

GOG 5 Gynecologic Oncology Group.

A3. ESMO-ESGO-ESTRO risk classificationFIGO 2009 staging used; molecular factors were considered but not included; tumour size was considered but not included; nodal status may be considered for treatment recommendations.; LVSI, lymphovascular space invasion

## Appendix B - Competency Framework

Adapted from Guy's and St Thomas’ NHS Foundation Trust, London, United Kingdom

*Creutzberg CL, van Putten WL, Koper PC, et al. Surgery and postoperative radiotherapy versus surgery alone for patients with stage-1 endometrial carcinoma: Multicentre randomised trial. PORTEC Study Group. Post Operative Radiation Therapy in Endometrial Carcinoma. *Lancet* 2000; **355**: 1404–11.

Creutzberg CL, Van Putten WLJ, Koper PC, et al. Survival after relapse in patients with endometrial cancer: Results from a randomized trial. *Gynecol Oncol* 2003; **89**: 201–9.

Keys HM, Roberts JA, Brunetto VL, et al. A phase III trial of surgery with or without adjunctive external pelvic radiation therapy in intermediate risk endometrial adenocarcinoma: A Gynecologic Oncology Group study. *Gynecol Oncol* 2004; **92**: 744–51.

Nout RA, Smit V, Putter H, et al. Vaginal brachytherapy versus pelvic external beam radiotherapy for patients with endometrial cancer of high-intermediate risk (PORTEC-2): An open-label, non-inferiority, randomised trial. *Lancet* 2010; **375**: 816–23.

**Harkenrider MM, Block AM, Alektiar KM, et al. American Brachytherapy Task Group Report: Adjuvant vaginal brachytherapy for early-stage endometrial cancer: A comprehensive review. *Brachytherapy* 2017; **16**: 95–108.

Small W, Beriwal S, Demanes DJ, et al. American Brachytherapy Society consensus guidelines for adjuvant vaginal cuff brachytherapy after hysterectomy. *Brachytherapy* 2012; **11**: 58–67.

Hoskin P, Hellebust TP, Nout RA, et al. Endometrial cancer. In: E Van Limbergen, R Potter, P Hoskin D Baltas (eds). *The GEC ESTRO Handbook of Brachytherapy*, 2nd edn. European Society for Radiotherapy and Oncology, Brussels, Belgium, 2016; pp. 23.

*** Cameron AL, Cornes P, Al-Booz H. Brachytherapy in endometrial cancer: Quantification of air gaps around a vaginal cylinder. *Brachytherapy* 2008; **7**: 355–8.

Showalter T, Libby B, Ouhib Z. Avoiding incorrect site events in vaginal cylinder brachytherapy. BrachyBlast Email Newsletter Dec 2016. American Brachytherapy Society (ABS), Reston, VA.

## Appendix C – Statistical analysis

### C2: Descriptive Statistics – Weeks from Surgery to adjuvant VBT

#### Statistics

Weeks from Surgery to adjuvant VBT

### C.3 Descriptive Statistics – Time applicator in vaginal vault.

#### Statistics

Minutes applicator in vaginal vault

### C.4 Independent *t*-test − waiting time from surgery to treatment

Independent Samples Test

### C.6 Kaplain meir analysis: time to event of locoregional recurrence

Case Processing SummarySurvival Table
